# Stabilizing Off-pathway Oligomers by Polyphenol Nanoassemblies for IAPP Aggregation Inhibition

**DOI:** 10.1038/srep19463

**Published:** 2016-01-14

**Authors:** Praveen Nedumpully-Govindan, Aleksandr Kakinen, Emily H. Pilkington, Thomas P. Davis, Pu Chun Ke, Feng Ding

**Affiliations:** 1 Department of Physics and Astronomy, Clemson University, Clemson, SC 29634, USA; 2 ARC Centre of Excellence in Convergent Bio-Nano Science and Technology, Monash Institute of Pharmaceutical Sciences, Monash University, 381 Royal Parade, Parkville, VIC 3052, Australia; 3 Department of Chemistry, University of Warwick, Gibbet Hill, Coventry, CV4 7AL, United Kingdom

## Abstract

Experimental studies have shown that many naturally occurring polyphenols have inhibitory effect on the aggregation of several proteins. Here, we use discrete molecular dynamics (DMD) simulations and high-throughput dynamic light scattering (DLS) experiments to study the anti-aggregation effects of two polyphenols, curcumin and resveratrol, on the aggregation of islet amyloid polypeptide (IAPP or amylin). Our DMD simulations suggest that the aggregation inhibition is caused by stabilization of small molecular weight IAPP off-pathway oligomers by the polyphenols. Our analysis indicates that IAPP-polyphenol hydrogen bonds and π-π stacking combined with hydrophobic interactions are responsible for the stabilization of oligomers. The presence of small oligomers is confirmed with DLS measurements in which nanometer-sized oligomers are found to be stable for up to 7.5 hours, the time frame within which IAPP aggregates in the absence of polyphenols. Our study offers a general anti-aggregation mechanism for polyphenols, and further provides a computational framework for the future design of anti-amyloid aggregation therapeutics.

Aberrant aggregation of proteins into insoluble amyloid fibrils is implicated in a number of diseases including Alzheimer’s, Huntington’s and Parkinson’s diseases, and type-2 diabetes (T2D)^1^. Even though the aggregating proteins associated with these diseases are diverse in terms of their sizes, primary, secondary and tertiary structures, the corresponding amyloid aggregates display common features such as the characteristic cross-β structure of fibrils and cytotoxicity caused by either fibrils or intermediate oligomers, suggesting a common amyloid mechanism^1^. Among known amyloidogenic proteins and peptides, T2D-associated human islet amyloid polypeptide (hIAPP, a.k.a. amylin) is one of the most aggregation-prone peptides, which spontaneously forms amyloid aggregates *in vitro* within hours at μM concentrations^2^. IAPP is co-synthesized, co-stored and co-secreted with insulin by pancreatic β-cells, and has functions related to the control of gastric emptying and glucose intake^3^,^4^. Accumulating experimental evidence suggests that the amyloid aggregates of hIAPP are associated with β-cell death in T2D. Hence, inhibition of aggregation is a promising therapeutic approach against T2D as well as other amyloid diseases^5^,^6^.

Amyloid aggregation is initiated with the nucleation of aggregation seeds from unfolded or unstructured monomers and subsequently proceeds rapidly with the elongation of amyloid fibrils. Therapeutic strategies have been designed to target various species along the amyloid aggregation pathway. For example, proteins such as transthyratin (TTR) and superoxide dismutase 1 (SOD1) form native homo-oligomers; stabilization of these functional oligomers by small molecule binding reduces the availability of protein monomers necessary for amyloid aggregation formation^6^,^7^,^8^. Similarly, stabilization of protein monomers from misfolding and self-association can effectively prevent amyloid aggregation, which can be achieved by binding with ligands of proteins, antibodies, small molecules or ions^9^,^10^. Based on the hypothesis that soluble oligomers rather than insoluble amyloid fibrils are toxic, small molecules promoting fibril formation have been exploited to reduce amyloid cytotoxicity^11^. Molecules, which form colloid aggregates and sequester protein monomers to form large non-amyloid aggregates, have been categorized as colloidal inhibitors^12^.

Another attractive set of amyloid aggregation inhibitors are small molecule polyphenols such as EGCG^13^, curcumin^14^,^15^, and resveratrol^16^, which reduce aggregation and the related cytotoxicity of not only hIAPP^14^,^15^,^17^,^18^,^19^,^20^,^21^,^22^,^23^ but also other proteins and peptides such as amyloid- β^24^,^25^,^26^,^27^. Polyphenols have the advantage that they are naturally occurring, and non-toxic at moderate concentrations. Despite the known therapeutic benefits of small molecules^28^, pharmacological use of these polyphenols is often limited due to some common issues, such as their low water solubility and toxicity at high concentrations. Furthermore, the molecular mechanism of their anti-aggregation effect remains elusive, preventing the design of new or modified drug candidates. Therefore, it is highly valuable to uncover the inhibition mechanisms of these anti-amyloid small molecule polyphenols.

Several studies have focused on understanding the anti-aggregation mechanisms of small molecule polyphenols. For example, ion mobility spectrometry-mass spectrometry experiments showed that EGCG exerted an inhibitory effect on hIAPP aggregation, through direct binding of EGCG to the peptide^20^ and formation of off-pathway oligomers^13^. Computational modeling has also been applied to bridge the gaps of time and length scales between experimental observations and the underlying molecular systems. Using replica exchange simulations of the amyloidogenic segment of hIAPP, resveratrol was found to bind and prevent the lateral growth of the fibril-like β-sheets^29^. In another work, resveratrol was found to bind weakly to IAPP and reduce inter-peptide contacts^30^. A recent computational study showed that resveratrol altered the structure of an hIAPP pentamer^31^, which was modeled by the amyloid fibril structure derived from solid state NMR^32^. Despite multitudes of these studies, the molecular mechanism of inhibition remains largely unknown.

In this work, we applied atomistic discrete molecular dynamics (DMD) simulations to investigate the molecular mechanism of the inhibitory effects of two anti-aggregation molecules: curcumin and resveratrol. Interestingly, an early experimental study reported that aspirin, a structural derivative of phenol, had an inhibitory effect on IAPP aggregation^33^, however, no inhibition by aspirin was found in later studies by two different groups^20^,^34^. As a control, we also studied the effect of aspirin on hIAPP aggregation. DMD simulations feature rapid sampling of conformational dynamics and also accurate modeling of interactions important for protein folding and aggregation^35^,^36^, allowing the examination of large molecular systems with long time scales. We have employed a multiscale approach by combining atomistic and coarse-grained DMD simulations to study the protofibril formation of SOD1^37^. Recently, we have employed DMD simulations to model the self-association and oligomer formation of IAPP and to study the effects of insulin and ions on hIAPP aggregation (ref 9. and Nedumpully-Govindan *et al*., unpublished data). Here we performed unbiased DMD simulations of molecular systems consisting of up to eight hIAPP monomers and sixteen small molecules, and showed that the polyphenols stabilized small hIAPP clusters via nanoscale assembly, thereby preventing or slowing down further aggregation of hIAPP. Our predictions were validated by direct solution measurements using high-throughput dynamic light scattering (DLS), where resveratrol was found to stabilize nano-sized hIAPP/resveratrol complexes, inhibiting amyloid aggregation. In our DMD simulations, we found that the nanoassemblies were consisted of a hydrophobic core of polyphenol molecules structurally reinforced by surrounding proteins. The clusters were stabilized by π-π stacking of the aromatic groups of the proteins and polyphenol molecules as well as hydrogen bonds between the hydroxyl groups of the polyphenols and the protein backbones or sidechains. Our study has revealed, for the first time, the atomistic details of inhibitor-induced off-pathway oligomer formation of amyloid proteins. This new insight may prove highly beneficial to the rational design of alternative nanoassembly inhibitors.

## Results

### Self-assembly of small molecules

We first simulated the self-assembly of small molecules by randomly placing 16 aspirin, curcumin or resveratrol molecules in a box (see Methods for details). Ten independent simulations with different initial conditions were performed. We found that the two polyphenols, curcumin and resveratrol, self-assembled into large aggregates while aspirin favored to remain as monomers (Fig. S1). We analyzed the steady states reached by the three species, and found that the probability of finding a monomer (Fig. S1B) is the highest for aspirin, followed by resveratrol and curcumin. This is in the same order as the solubility of the small molecules. Specifically, aspirin is highly water soluble (solubility ~17 mM), whereas curcumin and resveratrol possess solubility of ~1.6 μM^38^ and ~131 μM^39^, respectively. The probability of finding a 16-mer is, however, also higher for resveratrol than curcumin, which is zero for aspirin.

#### Curcumin and resveratrol show high propensity to IAPP monomers than aspirin

**Figure 1:**
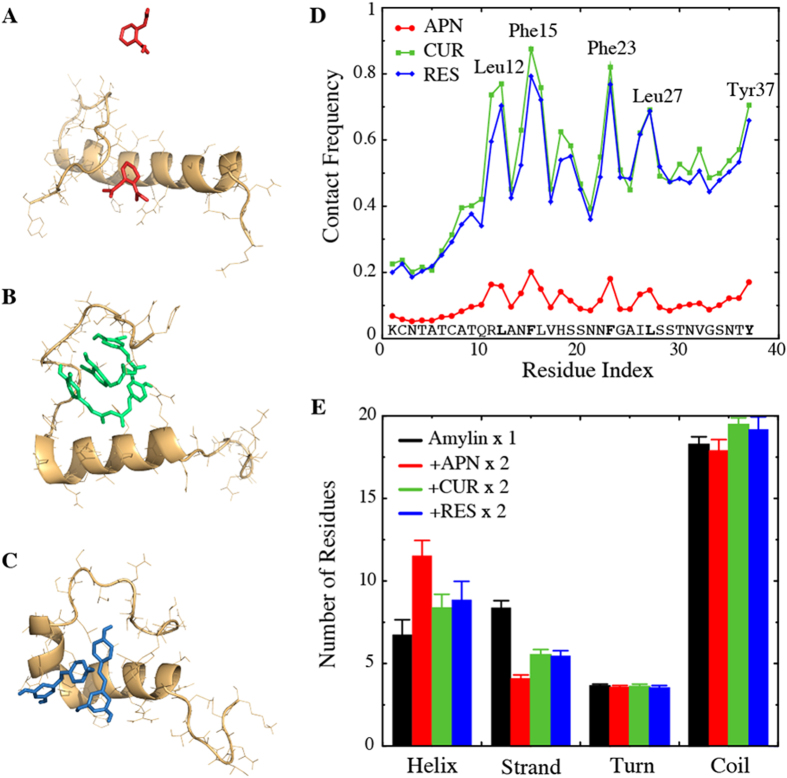
Interaction of small molecules with IAPP monomer. (**A–C**) Representative snapshots showing the final structures from simulations with (**A**) aspirin, (**B**) curcumin and (**C**) resveratrol. (**D**) Contact frequency of small molecules with IAPP residues. The IAPP-small molecule contact is dominated by the hydrophobic residues. (**D**) Small molecules stabilize helices while the β-strand content of IAPP is reduced.

We next simulated systems consisting of a single IAPP peptide and two small molecules. The effective ratio of the small-molecules to IAPP typically varied from 1 to 10 in experiments^20^, and hence we chose an intermediate ratio of 2 in our simulations. Examination of the simulation trajectories indicated that the binding behavior of aspirin with IAPP was different from that of polyphenols curcumin and resveratrol. Specifically, the two individual polyphenol molecules were often paired with each other and strongly bound to the peptide (representative snapshot structures of the simulations in Fig. 1A,B). However, aspirin molecules showed a very low affinity for binding with each other or with the peptide (Fig. 1C). The observed differential binding with IAPP by different small molecules is probably because of the difference in hydrophobicity. Due to their relatively high hydrophobicity, curcumin and resveratrol are likely to self-associate or bind to the hydrophobic residues of the peptide. To validate this hypothesis, we calculated the average number of atomic contacts (Supplementary Fig. S2) and contact frequency (Fig. 1D) of the small molecules with each IAPP residue. Examination of simulations indicated that all hIAPP monomer simulations reached their equilibrium or steady states rapidly (~10 ns) due to the small system sizes (data not shown). As discussed later, even the largest simulated systems reached their steady states by half of the simulation time (~25 ns). Hence, for consistency all reported averaging were carried out over the last half of trajectories, and over ten independent simulations. Two atoms within 5.5 Å were counted as an atomic contact, while a residue having at least one atomic contact with a ligand was defined as a contact. We found that the two polyphenols indeed showed high probability for binding the hydrophobic residues Leu12, Phe15, His18, Phe23, Leu27 and Tyr37. In comparison, aspirin made considerably fewer contacts with the protein than either curcumin or resveratrol. We also calculated the total peptide-binding time of the small molecules and found that, while aspirin bound ~57% of the time, the polyphenols bound the peptide during ~99% of the time. The small size and low affinity of aspirin to bind IAPP as a dimer may also have contributed to its particularly low binding frequency as shown in Fig. 1D. Even though aspirin’s affinity to bind IAPP residues was smaller than that of polyphenols, the dynamic binding with residues 10–30 (Fig. 1D) increased the corresponding probability to form helices (Fig. 1A,E).

We further examined the secondary structure contents of the peptide in the presence and absence of small-molecule binding (Fig. 1E). In the control simulations without any small molecules, the peptide was mostly unstructured with about 7 residues forming an α-helix and 11 residues forming a β-strand, in agreement with previous *in vitro* and computational studies^2^,^9^,^40^. An increase in the helix content and decrease in the β-strand content were observed in the presence of all three types of small molecules. Even though binding of aspirin to IAPP was weaker than polyphenols, it exerted the strongest influence on the α-helix and β-strand contents of the peptide. No significant changes of the turns or coils were caused upon small molecule binding. To evaluate the formation of tertiary structures, we also computed residue pair-wise contact frequency map of IAPP monomer with and without small-molecule binding (Fig. S3). In the absence of ligands, IAPP monomer did not have significant long-range tertiary contacts, except the disulfide-bond related interactions at the N-terminal and a weak antiparallel interaction with a turn near residues 20–23 (Fig. S3A). The weak long-range antiparallel interaction was disrupted by all three small molecules (Fig. S3B–D).

#### Curcumin and resveratrol reduce self-association of IAPP

**Figure 2:**
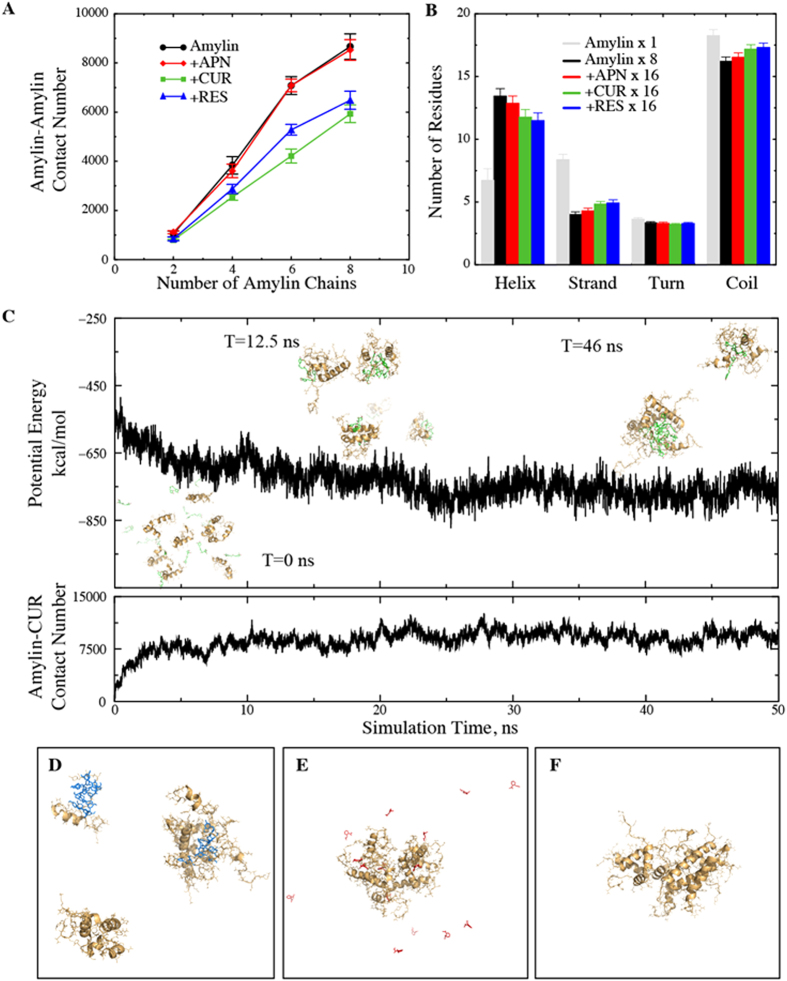
Interactions of small molecules with multiple IAPPs. (**A**) IAPP-IAPP contact number as a function of number of IAPP chains. The contact number is smaller in the presence of curcumin and resveratrol, while aspirin has no apparent effect. (**B**) Secondary structure contents for the octamer IAPP system. The overall number of helical residues is increased in comparison with the monomer IAPP system. Also, the small molecules have an opposite effect on the helical and strand contents of IAPP. (**C**) Typical trajectories of potential energy and IAPP-curcumin contact number for the octamer IAPP system. Snapshots of the system at different times are shown in the inset of (**C**). Representative final snapshots of IAPP systems with (**D**) resveratrol, (**E**) aspirin and (**F**) no ligand.

To understand the effect of small-molecule binding on the self-association of multiple peptides, we performed DMD simulations with 2, 4, 6 and 8 IAPPs, where the IAPP to small molecule ratio (1:2) and the peptide density was kept unchanged from those in the monomer simulation. Since the amyloid aggregates are rich in inter-peptide β-sheets, we expect the probability of amyloid nucleation to be correlated with the number of inter-chain contacts. Thus, for each set of multi-IAPP simulations, we calculated the total inter-chain IAPP contacts averaged over ten independent trajectories (Fig. 2A). As expected, as the number of IAPPs increased, the total number of inter-peptide contacts increased. The contact numbers in IAPP-aspirin binding simulations were indistinguishable from the control simulations (peptides alone), suggesting that aspirin had little effect on IAPP self-association. Along with the weak affinity of aspirin for IAPP monomer (Fig. 1), our simulation results are consistent with the latest experimental observation that aspirin had little inhibitive effect on IAPP aggregation^20^,^34^. In contrast, in the presence of curcumin and resveratrol the peptide chains made fewer contacts with each other. Also, curcumin displayed a stronger effect on reducing IAPP-IAPP contacts than resveratrol. We also calculated the number of atomic contact between amyloidogenic residues of IAPP, residues 22–29 (Supplementary Fig. S4). This segment alone can form fibrils *in vitro*, and is believed responsible for the high amyloidogenic propensity of human IAPP^41^,^42^. Similarly, the amyloidogenic contact number was also smaller in the presence of polyphenols in comparison with simulations of peptide alone or peptide with aspirin binding (Supplementary Fig. S4). Hence, DMD simulations suggest that polyphenols inhibit IAPP aggregation by a reduction of the inter-peptide association, thereby reducing the probability for the peptides to nucleate the formation of fibrils, while aspirin did not show this effect.

To evaluate whether the binding of small molecules leads to any structural changes of the peptides, we first calculated the secondary structure contents per IAPP chain with and without small molecules binding (e.g. Fig. 2B, simulation of eight IAPPs). Firstly, in the absence of small molecules, the peptides were significantly more helical than IAPP monomers. This observation is in agreement with several experimental studies in which α-helix rich intermediate states have been reported^42^,^43^,^44^,^45^. The presence of helical intermediates have also been observed en route to the aggregation of a number of other peptides and it has been hypothesized that helix-helix interaction plays a key role in aggregation^46^. The helices formed by IAPP were amphiphilic and only marginally stable in solution (helical propensity of each residues shown in Supplementary Fig. S5). In the absence of small molecules, the associations between such helices stabilized them to increase the helix content. Binding of small molecules with peptide monomers had a similar stabilizing effect of helices, but to a lesser extent (Fig. 1E). In the presence of both aspirin and polyphenols, we found that relatively smaller number of residues formed helices (Fig. 2B). Our results suggested that the peptides in the IAPP-small molecule complexes were likely stabilized by the interactions between peptides and small molecules rather than peptide-peptide interactions. We calculated the inter-peptide contact frequency between all pair-wise residues (Fig. S6). In the absence of small molecules, the inter-peptide quaternary interactions were dominated by the N-terminal residues near Phe15 and His18 and the amyloidogenic region (Fig. S6A). Consistent with the total number of inter-peptide contact number, aspirin showed no significant effect on contact map (Fig. S6B). In the presence of curcumin and resveratrol, the overall quaternary contact was reduced (Fig. S6C–D). Especially, the inter-peptide interactions mediated by residues Phe15 and His18 were lost, which instead formed interaction with the polyphenols. Our results highlight the importance of N-terminal aromatic residues Phe15 and His18 for the self-association and aggregation of IAPP.

#### Polyphenols engender nano-sized clusters of IAPPs

To address how polyphenols reduce the total inter-peptide contacts, we examined the simulation trajectories (e.g. a simulation of eight IAPPs with 16 curcumin molecules in Fig. 2C). As the simulation progressed, the initially separated peptides and curcumins started to aggregate and form a few clusters (see the inset of Fig. 2C). Similarly, simulations with resveratrol also often resulted in multiple IAPP-ligand clusters, each consisting of a few peptides (Fig. 2D). In contrast, in the absence of polyphenols (peptides alone or with aspirin) the peptides aggregated into a single large cluster (Fig. 2E,F), in agreement with a previous experimental study^47^. Therefore, our results indicate that polyphenols can reduce the total number of contacts between IAPPs by forming smaller clusters.

**Figure 3:**
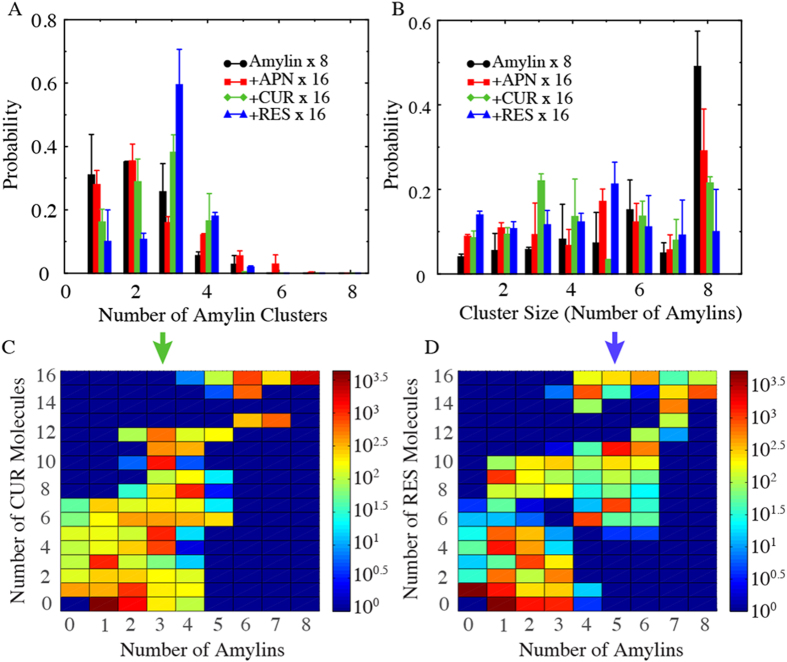
Clustering properties of the octamer IAPP system. (**A**) The probability of the number of IAPP-containing clusters. For IAPP alone and IAPP-aspirin systems one or two clusters are most populated while three clusters is most populated for IAPP-resveratrol and IAPP-curcumin systems. The error bars correspond to standard deviations from independence simulations. (**B**) Histogram of the cluster size. Three and five IAPP-containing clusters are most populated for curcumin- and resveratrol-IAPP systems, while eight IAPP-containing cluster is the most common one for IAPP alone and IAPP-aspirin systems. (**C,D**) Compositions heat map for IAPP-curcumin and IAPP-resveratrol clusters.

To characterize these IAPP clusters formed in the simulations, we first identified the protein-containing clusters from DMD simulations of eight IAPP peptides. A peptide molecule or a small molecule belonged to a cluster if it made at least one atomic contact with a member molecule within the cluster. For all simulations, we used the second half of all independent simulations (where the systems were already equilibrated as shown by the time evolution of potential energies and inter-molecular contacts in Fig. 2C and number of clusters in Fig. S7) and characterized the clustering behavior by calculating the number and size of clusters, as well as the composition of each cluster. We first computed the histograms of the number of clusters (Fig. 3A). In the absence of small molecules, eight IAPP chains tended to aggregate into 1-2 clusters. In the presence of aspirin, IAPP displayed a very similar aggregation profile, again suggesting a weak effect of aspirin on IAPP aggregation. In the presence of resveratrol and curcumin, the IAPPs started to form more clusters. Especially with resveratrol, the IAPPs displayed a high preference to form three clusters.

Additionally, we computed the mass-weighted histogram of the cluster size (the number of protein chains in the cluster), which estimated the probability of each peptide to form a cluster of a given size (Fig. 3B). In the absence of small molecules, peptides alone tended to aggregate into a single octamer cluster. A second peak corresponding to an IAPP hexamer cluster was also observed but was significantly smaller than the octamer peak. Similarly, an IAPP octamer was also the dominant cluster in the presence of aspirin, followed by an IAPP pentamer. Noticeably, in the presence of resveratrol a peptide showed the highest probability to aggregate into pentamers, while in the presence of curcumin clusters consisting of three IAPPs were the most common, which is closely followed by the octamer. To further characterize these clusters induced by polyphenols, we analyzed their compositions, i.e., the numbers of peptides and small molecules. We calculated the heat-map of clusters denoted by the number of small molecules versus the number of peptides (Fig. 3C,D). We found that there was an overall ratio of 2:1 between small molecules and peptides for the clusters, which is determined by the molar ratio used in the simulations. On the other hand, analysis indicates that the oligomers were molecular complexes composed of both small molecules and peptides. The clustering analysis of our simulations suggests that polyphenols curcumin and resveratrol enabled the formation of stable oligomers composed of a relatively small number of peptides and small molecules, in drastic contrast to the aggregate state of large clusters by peptides alone.

#### Polyphenols form the core while IAPPs participate in the “corona” of the nano-assembled oligomer

**Figure 4:**
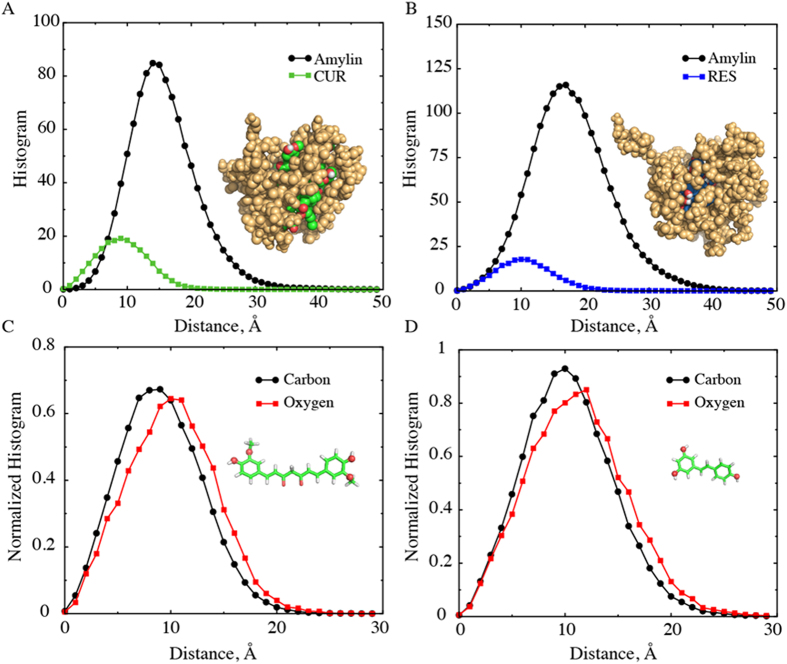
Properties of stable small molecule-IAPP clusters. (**A**,**B**) Radial distributions of IAPP and small molecule atoms from the center of mass of the cluster, for (**A**) curcumin-IAPP and (**B**) resveratrol-IAPP clusters. Typical structures of the clusters are shown in inset; protein atoms in golden-yellow, curcumin in green (carbon) and red (oxygen), and resveratrol in blue (carbon) and red (oxygen). (**C,D**) Distributions of small aliphatic carbon and oxygen atoms of curcumin and resveratrol inside the clusters. (Inset) Structures of curcumin and resveratrol, highlighting the oxygen atoms used for calculations as spheres.

We characterized the most stable clusters formed in the presence of small molecule phenols, namely IAPP trimers for curcumin and IAPP pentamers for resveratrol (Fig. 3B). We isolated these oligomers from the simulation trajectories and generated the corresponding ensembles. Representative structures of small-molecule-induced oligomers (inset of Fig. 4A,B) suggest that the small molecules were positioned inside and peptides outside. To verify this observation, we computed the radial distribution of the peptide and small molecule atoms as a function of the distance from the center of mass of a cluster, averaged over the structural ensemble of corresponding oligomers (Fig. 4A,B; and the corresponding density profiles in Supplementary Fig. S8). Indeed, in both cases, the small molecules were buried inside the oligomer with its peak 9–10 Å away from the center of mass, while the distribution of the protein atoms was shifted to the right. The peak of protein atom distribution is 14–15 Å for the curcumin-binding-induced trimers, and 16-17 Å for the resveratrol-induced pentamers. We can also see from the insets of Fig. 4A,B that many hydroxyl groups of the polyphenols were exposed (red spheres in Fig. 4A,B). Therefore, we further computed the radial distributions of aliphatic carbon and oxygen atoms of the small molecules inside the oligomer (Fig. 4C,D). We found that the aliphatic carbons of both curcumin and resveratrol were distributed closer to the core than the hydrophilic oxygen atoms. Thus, the small molecule polyphenols inside the cores of the oligomers had their hydrophobic atoms buried inside and hydrophilic hydroxyl groups outside. In comparison, in the presence of aspirin the IAPP octamer (Fig. 2E, corresponding to the highest peak in Fig. 3B) and pentamer structure (e.g., a snapshot structure in Fig. S9, corresponding to second peak in Fig. 3B) had no core formed by small molecules. Aspirin molecules either stayed unbound or bound individually to the surface of oligomers.

#### Dynamic light scattering measurements

**Figure 5:**
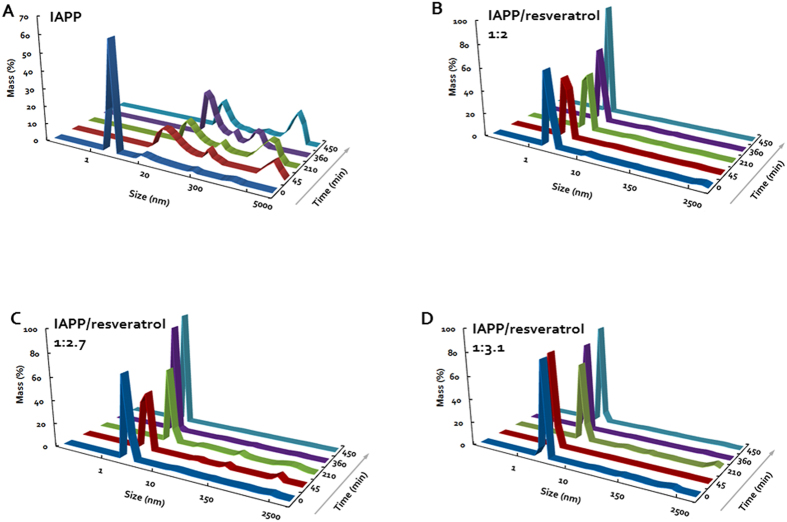
Time evolution of the hydrodynamic diameters of IAPP (**A**) and IAPP-resveratrol at 1:2, 1:2.7 and 1.3.1 molar ratios (**B–D**). IAPP concentration in all samples: 19 μM.

Other experimental studies have reported the existence of small spherical IAPP aggregates in the presence of resveratrol using surface measurements by atomic force microscopy (AFM)^17^,^48^. The sizes of these structures were consistent with the small molecular weight oligomers observed in our DMD simulations. For proof of concept we carried out an independent solution measurement using high-throughput DLS to characterize the size distribution of IAPP in the presence and absence of resveratrol. Curcumin was not included in the DLS experiment due to its much poorer water solubility, which is two orders of magnitude lower than that of resveratrol. As expected, the DLS measurement revealed a rapid assembly of IAPP at 19 μM in Milli-Q water into large fibrils over a time course of 450 min (Fig. 5A). Specifically, at time zero when the experiment just began, the IAPP control displayed a pronounced peak value (86.5% mass) of hydrodynamic diameter at 2.2 ± 0.7 nm, approximately the size of a monomer, accompanied by a small peak at 30.4 nm (10.5% mass). At the time point of 45th min, the most pronounced peak of the protein (66.2% mass) was shifted and broadened to 31.3 ± 3.2 nm, alongside two other major peaks at 238.5 nm (12.5% mass) and 5,936.5 nm (21.4% mass), respectively. Further incubation of the protein at room temperature saw its major peaks rose to 46.3 ± 3.7 nm (49.5% mass) and 2,766.4 nm (44.2% mass) at time point of 210th min, 60 ± 6.6 nm (74.4% mass) and 761.5 nm (19.3% mass) at time point of 360 min, and 65.1 ± 4.9 nm (44.8% mass) and 3,451.1 ± 93.6 nm (47.5% mass) at time point of 450 min, indicating an irreversible process of amyloid fibrillation.

In the presence of resveratrol, however, the process of IAPP fibrillation came to a halt, as clearly shown by the evolution of the protein hydrodynamic diameter in Fig. 5B–D. The resveratrol control at 39 μM registered no distinct peaks as expected, ensuring repeatability of the DLS experiment. In contrast to the IAPP control, the single and major peak of the hydrodynamic diameter ranged between 1.7~2.7 (±0.5) nm, 1.5~4.2 (±0.8) nm and 1.8~4.2 (±1.0) nm for the IAPP/resveratrol molar ratio of 1:2, 1:2.7 and 1:3.1, respectively (Fig. 4B–D). This result is in good agreement with the DMD observation that each IAPP pentamer encased multiple hydrophobic resveratrol molecules to render small clusters of (Figs 3,4 and Supplementary Fig. S4), thereby disrupting amyloid fibrillation. This result further suggests that excess supply of resveratrol – whose concentration was still below its solubility – did not significantly destabilize the IAPP-resveratrol clusters. These excess small molecules either stayed dissolved in the solution, or were partitioned into the hydrophobic cores of the IAPP pentamers or higher-order assemblies, as reflected by the comparable but slightly increased hydrodynamic diameters observed for IAPP/resveratrol molar ratios of 1:2.7 and 1:3.1 than 1:2.

## Discussions

A number of experimental studies have reported the inhibitory effect of curcumin and resveratrol on IAPP aggregation^14^,^15^,^17^,^18^, but the studies on the effect of aspirin on IAPP aggregation do not converge^20^,^33^,^34^. According to our atomistic DMD simulations, polyphenols curcumin and resveratrol bound IAPP monomers strongly and reduced self-association of IAPP by stabilizing IAPP oligomers of small molecular weights. On the other hand, aspirin showed weak binding to IAPP monomer and exerted little effect on the self-association of IAPPs. Therefore, our simulations support the scenario^20^,^34^ that aspirin has little inhibitory effect on IAPP aggregation. The reported inhibitory effect in the earlier study^33^ was probably due to the interference of aspirin with the Congo-red assay that was used to monitor amyloid fibril formation of IAPP, as noted in ref. 34.

For many amyloidogenic proteins such as amyloid-β and α-synuclein, experimental studies identified significant small oligomer intermediates populated along the aggregation pathway^49^,^50^,^51^,^52^. Our DMD simulations of multiple IAPP alone suggested that the peptides tend to form a single large aggregate (Fig. 3B). Despite a high peptide concentration (~ 6 mM) in our simulations, such a tendency of IAPP forming large aggregate was reported by previous NMR and sedimentation velocity studies^47^,^53^. Different groups reported the accumulation of α-rich intermediate states of IAPP before the formation of β-rich IAPP fibrils^42^,^43^,^44^,^45^. Our control simulations of multiple IAPPs captured the increase of α-helixes compared to the simulation of IAPP monomer (Fig. 2). The increase in helical content upon initial association was due to the stabilization of marginally stable helices by inter-chain interactions. In the presence of curcumin or resveratrol, IAPPs showed a reduction of helical content with a corresponding small increase in β-strand and random coil structures (Fig. 2). This observation is consistent with a reported experimental study of the effect of curcumin on IAPP aggregation^14^, where circular dichroism (CD) revealed the decrease of α-helix when IAPPs were incubated with curcumin. Therefore, our computational studies are able to recapitulate the secondary structure contents of IAPP under various experimental conditions.

Raleigh and coworkers applied point mutations to analyze the interaction of resveratrol with IAPP^18^. Accordingly, residues Phe15, His18 and Tyr37, and possibly Arg11 were the most important residues for resveratrol-IAPP interactions. According to our simulations, residues that make significant contact with resveratrol (and also with curcumin) were Arg11, Phe15, His18, Phe23, Leu27 and Tyr37 (Fig. 1D and Supplementary Fig. S1). Thus the simulations and mutagenesis studies converge on the roles of residues Arg11, Phe15, His18 and Tyr37, but not Phe23. Interaction of Leu27 with small molecules has not been studied in the experiment. As also noted by Tu *et al*.^18^, solid-state NMR structure of IAPP fibrils indicated that Phe23 was positioned in a loop connecting the two β-strands, while the other residues were positioned in the β-strands. Even if resveratrol bound Phe23, it may not exert a strong effect on the IAPP aggregation process, and hence mutation of this particular residue may not show any significant effect on the aggregation-inhibition by resveratrol.

In addition to mutagenesis assays, atomic force microscopy (AFM) has been used to study the morphology of IAPP aggregation. In the presence of resveratrol, AFM measurements reported the absence of IAPP fibrils, while detected the existence of small spherical structures with diameters of ~3–4 nm^17^, in agreement with our DLS measurement (Fig. 5B–D). Similar spherical structures have also been observed in a synchrotron X-ray reflectivity study in the inhibition of lipid membrane-induced IAPP aggregation by resveratrol^48^. Inhibition of protein aggregation by a colloidal mechanism — molecules form colloids and sequester proteins — has been reported in the past^12^,^20^. However, the sizes of colloids in this case are much larger than that of the small clusters of IAPP stabilized by polyphenols. In our DMD simulations, we observed the formation of small IAPP oligomers, which have a diameter of ~4 nm (see Fig. 4 and Supplementary Fig. S4), closely matching with the experimentally observed IAPP cluster radius. Although AFM might have limitations to capture the structures of these oligomers in solution, our computational and DLS studies together with the AFM measurements in the literature strongly support the formation of small IAPP oligomers in the presence small molecules.

**Figure 6:**
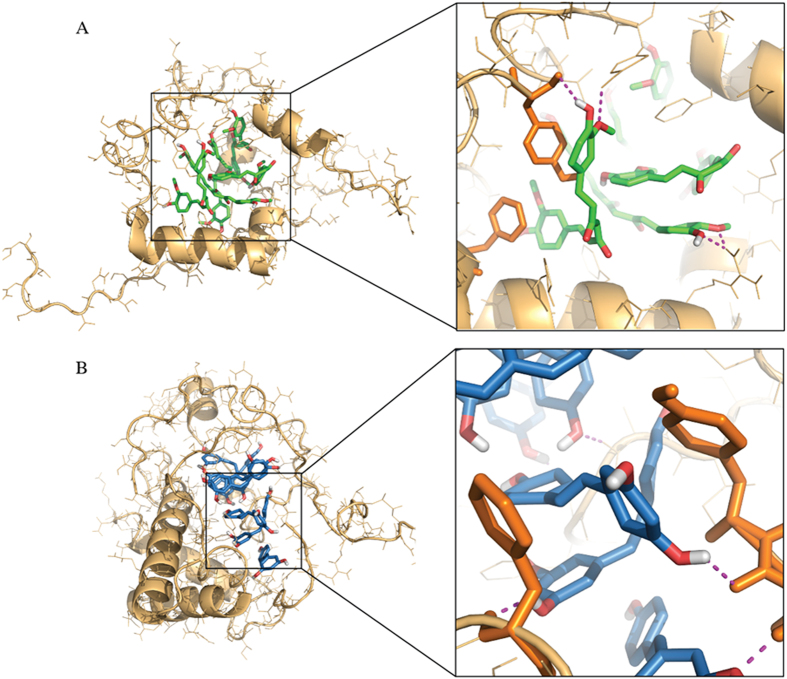
Stabilizing forces of small molecule-IAPP clusters. (**A**) Curcumin-IAPP and (**B**) resveratrol-IAPP clusters are stabilized by π-π stacking and hydrogen bonds. Zoomed-in snapshots depict stacking of phenyl groups of the small molecules against that of the protein. The hydroxyl groups of the small molecules make hydrogen bonds with the protein backbones and side chain atoms (shown using magenta dashed lines).

Many polyphenols have been found to have anti-aggregation effects on IAPP as well as some other amyloidogenic peptides, such as Aβ in Alzheimer’s disease. Our DMD simulations of two model molecules, curcumin and resveratrol, suggest a common mechanism – these polyphenols can stabilize the formation of off-pathway oligomers where the small molecules self-assemble in a nano-sized core and IAPP peptides bind to the peripheries. To evaluate how the small molecular clusters stabilized the formation of IAPP oligomers, we examined the interactions between the small-molecule core and the bound IAPP peptides (Fig. 6). Remarkably, the phenyl groups of the small molecules formed π-π stacking with the aromatic side chains of the peptides. In addition, we also observed extensive hydrogen bond formation between the hydroxyl groups of the polyphenols and the peptide backbones or side chains (magenta dashed lines in Fig. 6). Therefore, π-π stacking and hydrogen bonding, characteristic interactions associated with the phenol moiety, were important for the hetero-molecular complex formation. The aromatic side chains, especially Phe15, strongly influenced and contributed towards IAPP aggregation^42^,^54^. Our simulation of IAPP alone also highlighted the importance of Phe15 in the peptide self-association and aggregation (Fig. S6A). When the residue takes part in π-π stacking with polyphenols, inter-chain interactions of aromatic residues will be affected. In this study, we used a small IAPP:small molecule ratio of 1:2 (1:2 – 1:3.1, in experiments).

The aggregation inhibitors can be strongly affected by the inhibitor concentration, e.g. as shown in the case of amyloid-β^55^. Similarly, the relative and absolute concentrations of both IAPP and small molecules may influence the inhibition efficiency and mechanism as well as the structure and composition of oligomers. Here, we focused on the inhibition mechanisms by different types of small molecules. The concentration dependence of aggregation inhibition by small molecules required further systematic investigations both computationally and experimentally. Similarly, membrane disruption by amyloid aggregates is believed to be the prime cause for cytotoxicity^56^. It will be of great interest to study whether polyphenols also inhibit membrane disruption by IAPP.

## Conclusions

We applied atomistic DMD simulations to uncover the anti-aggregation mechanism of two polyphenol molecules, curcumin and resveratrol, which have been experimentally found to inhibit IAPP aggregation. For comparison, we also studied aspirin, whose anti-aggregation effect is not conclusive^20^,^33^,^34^. Our simulations suggest that both curcumin and resveratrol could bind IAPP monomer strongly, but aspirin had a weaker binding to the peptide. Further simulations with multiple peptides and small molecules revealed that the anti-aggregation effects of these polyphenols were not due to the stabilization of IAPP monomers as in the aggregation inhibition by insulins^9^,^42^, but resulted from stabilization of off-pathway oligomers, a conclusion corroborated by the high-throughput DLS measurement of IAPP exposed to resveratrol. In the control simulations with peptides alone and in the presence of aspirin, we found that the peptides were prone to continuously self-associate into large oligomers. In contrast, polyphenols like curcumin and resveratrol nucleated the formation of stable oligomers with smaller molecular weights, e.g. trimers and pentamers induced by curcumin and resveratrol, respectively. Detailed analysis suggested that multiple polyphenol molecules formed the core, which provided both hydrophobic surfaces and hydrogen binding acceptors and donors (hydroxyls in the phenol groups) for peptides to bind. By burying the hydrophobic residues and unsatisfied hydrogen bonds inside, the oligomers were stable and did not continuously grow as in the case of the simulations for the case of peptides alone. The difference between curcumin and resveratrol in their physicochemical properties, such as geometry, conformational flexibility, and molecular weight, determined the formation of distinct oligomers. Further studies combining DMD simulations and experimental characterizations will help design optimal anti-aggregation small molecules to inhibit IAPP aggregation in T2D.

## Methods

### DMD Simulations

Simulations were carried out using discrete molecular dynamics (DMD) algorithm^35^,^36^. In DMD the inter-atomic interactions have similar components as conventional molecular mechanics force fields, but the interactions are modeled using discrete potential functions mimicking the continuous potential functions. The simulations were carried out as a series of collisions, which occurred when a potential step was encountered by a pair of atoms. At such collisions, the atomic velocities were updated following the laws of conservations of energy, momentum and angular momentum. For modeling both proteins and small molecules, we employed Medusa force field^35^. The non-bonded inter-atomic interaction included van der Waals, solvation, hydrogen bond and electrostatic terms. The van der Waals parameters were adopted from the CHARMM force field^57^, and bonded termed were parameterized based on statistical analysis of protein structures from protein data bank (PDB) values. The water molecules were implicitly modeled using the EEF1 implicit solvation model developed by Lazaridis and Karplus^58^. A reaction-like algorithm was used to model hydrogen bonds^59^. The electrostatic interactions were screened using the Debye-Hückel approximation with screening length set to 10 Å, which corresponds to 100 mM of NaCl. The force field in all-atom DMD is physics-based and transferrable. The same force field has been shown to be able to accurately predict small-molecule ligand-receptor binding poses and estimate binding affinities^60^,^61^. Using all-atom DMD simulations, we were able to accurately model the structure and dynamics of polymeric PAMAM nanoparticles^62^ and their interactions with both linear and polyaromatic small molecule hydrocarbons^63^.

### Simulation Setup

The initial atomic structure of monomer IAPP was obtained from the protein data bank (PDB code 2L86^64^). The disulfide bond between residues 2 and 7 strongly influence the structure and aggregation of IAPP^32^,^65^. Hence we treated the disulfide bond as a covalent bond and kept it intact in simulations. We simulated different systems consisting of 1, 2, 4, 6 or 8 IAPP chains with two small molecules per IAPP chain. The simulations were performed in cubic boxes whose size was varied such that the density of IAPPs was approximately the same for all the systems. The sizes of the smallest (one IAPP) and largest (eight IAPPs) boxes were 63.7 Å and 127.3 Å, respectively. Simulations were set up by randomly placing IAPP(s) and small molecules inside the box. For the simulation of small molecules without IAPP, 16 small molecules were placed in a cubic box of size 127.3 Å. To improve sampling and remove any possible bias arising from the initial structure, ten independent simulations were carried out for each molecular system, in which the initial molecular positions, orientations, and atomic velocities were randomized using random number generation with different random seeds. The systems were first energy minimized for 1,000 steps using steepest descent algorithm, and then equilibrium simulations were carried out for one million DMD steps, which corresponds to a simulation time of ~50 ns. The temperature of the system was maintained at ~300K using Anderson thermostat^66^. Our analysis showed that the simulations reached steady state after about 25 ns, so the averaging of quantities was done over the last half of simulations.

### Clustering

The clustering analysis of IAPP and small molecules was based on pairwise contacts between molecules. At any instance, two molecules were defined to be in contact if the distance between any two inter-molecular atoms was smaller than 5.5 Å. If two molecules were directly in contact, or they were connected through contacts with one or more other molecules, they were defined to be in the same cluster.

### Dynamic Light Scattering Measurement

Human IAPP (KCNTATCATQRLANFLVHSSNNF GAILSSTNVGSNTY; modifications: Tyr-37 – C-terminal amide, disulfide bridge between cysteines 2 and 7; C165H261N51O55S2; MW: 3,903; purity: >95%) was purchased in lyophilized powder form from Abcam (www.abcam.com). Resveratrol was obtained in lyophilized powder form from Sigma-Aldrich (purity: 99%). The resveratrol was first dissolved in ethanol to 10 mg/mL and further diluted in Milli-Q water to reach a stock concentration of 0.02 mg/mL or 87 μM, well below the known resveratrol water solubility of 131 μM. The IAPP was weighed on a Cubis MSE (Sartorius) balance with 0.01 mg resolution and then dissolved in Milli-Q water to form a 0.25 mg/mL (64 μM) stock solution immediately prior to the DLS measurement.

#### High-throughput DLS

The hydrodynamic sizes of IAPP and IAPP-resveratrol mixtures were acquired at room temperature using an automated, high-throughput DLS device (DynaPro Plate Reader, Wyatt; instrument resolution: 0.5 nm) and black 384-well plates (Thermo Fisher). For each sample well, the volume of the protein was fixed at 6 μL while the volume of resveratrol was varied from 0 μL (IAPP control) to 9 μL, 12 μL and 14 μL. Each sample volume was then topped up to 20 μL with Milli-Q water, rendering an IAPP/resveratrol molar ratio of 1:2, 1:2.7 and 1:3.1, respectively. In addition, the resveratrol control (39 μM) constituted 9 μL of the small-molecule stock and 11 μL of Milli-Q water. The selection of these sample conditions ensured that the final IAPP concentration in each sample well (19 μM) was favorable for amyloid aggregation, resveratrol (up to 59 μM) was well dissolved, and the signals were sufficiently strong for detection. Prior to the measurement, the samples were spun for 1 min at 1,000 rpm/164 RCF (Centrifuge 5804, Eppendorf) to ensure good mixing. An optical module collected 20 data points for each sample well while scanning through all samples continuously. The acquired data were automatically processed and displayed using the Dynamics 7.1.7 software. To ensure repeatability and statistics each sample condition was measured in quadruplicate. The experiment ran for 450 min in total.

## Additional Information

**How to cite this article**: Nedumpully-Govindan, P. *et al*. Stabilizing off-pathway oligomers by polyphenol nanoassemblies for IAPP Aggregation Inhibition. *Sci. Rep.* **6**, 19463; doi: 10.1038/srep19463 (2016).

## Supplementary Material

Supplementary Information
