# Supplementary material for: Stabilizing Off-pathway Oligomers by Polyphenol Nanoassemblies for IAPP Aggregation Inhibition
Source: Sci Rep. 2016 Jan 14;6:19463. doi: 10.1038/srep19463 (PMC4725907; doi:10.1038/srep19463)
Supplement: Supplementary Information [file srep19463-s1.pdf]

Supplementary Information

Stabilizing off-pathway oligomers by polyphenol nanoassemblies for IAPP aggregation inhibition

Praveen Nedumpully-Govindan,[1] Aleksandr Kakinen,[2] Emily H. Pilkington,[2] Thomas P. Davis,[2,3] Pu Chun Ke,[2,*] Feng Ding[1,*]

[1]Department of Physics and Astronomy, Clemson University, Clemson, SC 29634, USA

[2]ARC Centre of Excellence in Convergent Bio-Nano Science and Technology, Monash Institute of Pharmaceutical Sciences, Monash University, 381 Royal Parade, Parkville, VIC 3052, Australia

[3]Department of Chemistry, University of Warwick, Gibbet Hill, Coventry, CV4 7AL, United Kingdom

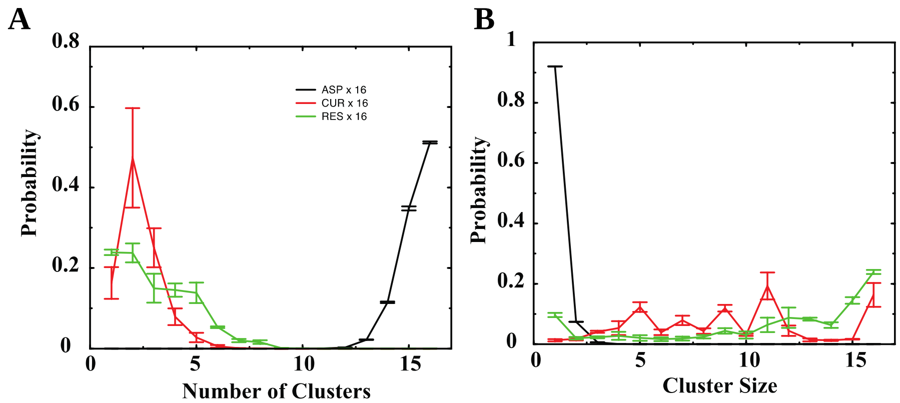

Fig. S1. The self-assembly of small molecules. (A) The distribution of number of clusters and (B) cluster sizes of aspirin, curcumin and resveratrol.

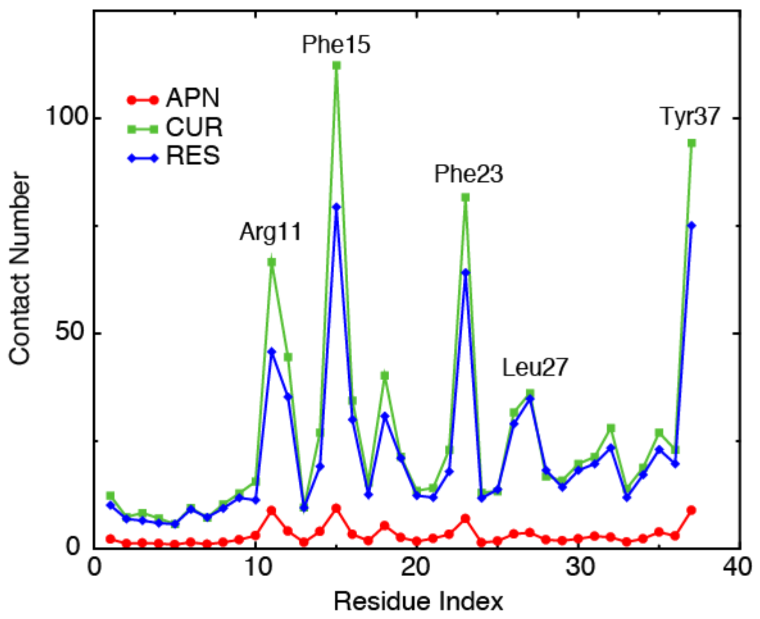

Figure S2. Interactions of small molecules with IAPP. Average small molecule contact number for each of peptide residues from DMD simulations of two small molecules with one IAPP monomer. Aspirin makes fewer contacts than the polyphenols curcumin and resveratrol. The peptide residues with largest contact numbers are mostly hydrophobic.

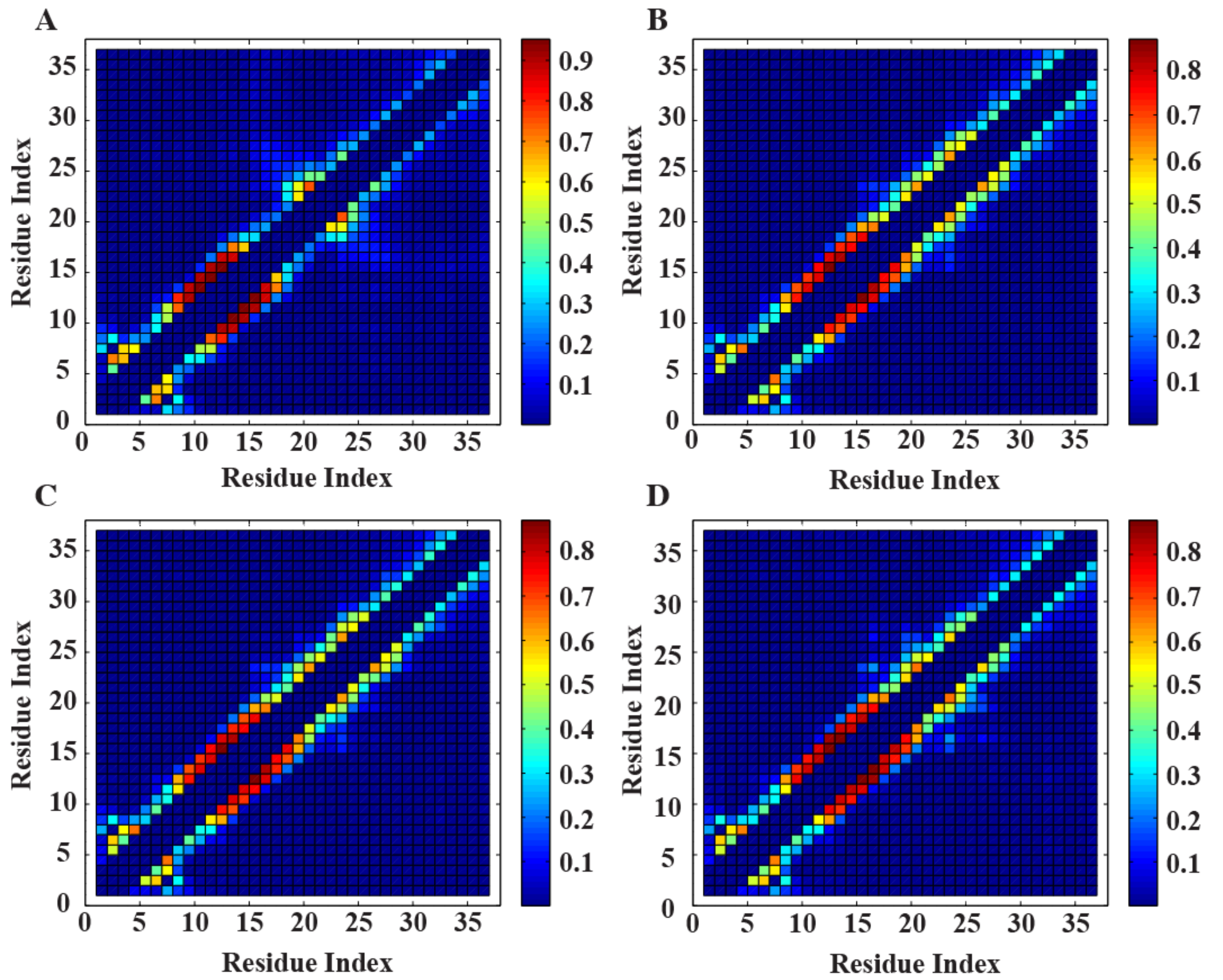

Fig. S3. Tertiary structure of IAPP. The amino acid contact frequency maps of IAPP in the absence of small molecules (A), and in the presence of (B) aspirin, (C) curcumin and (D) resveratrol molecules. The contacts between alpha carbon atoms of amino acids, with a cutoff of 7.5 Å, were used to calculate the contact frequency. The short-range contacts along the backbone, i.e. nearest and next-nearest contacts near the diagonal and the disulfide-bond between residues 2 and 7 that have permanent contacts, were excluded.

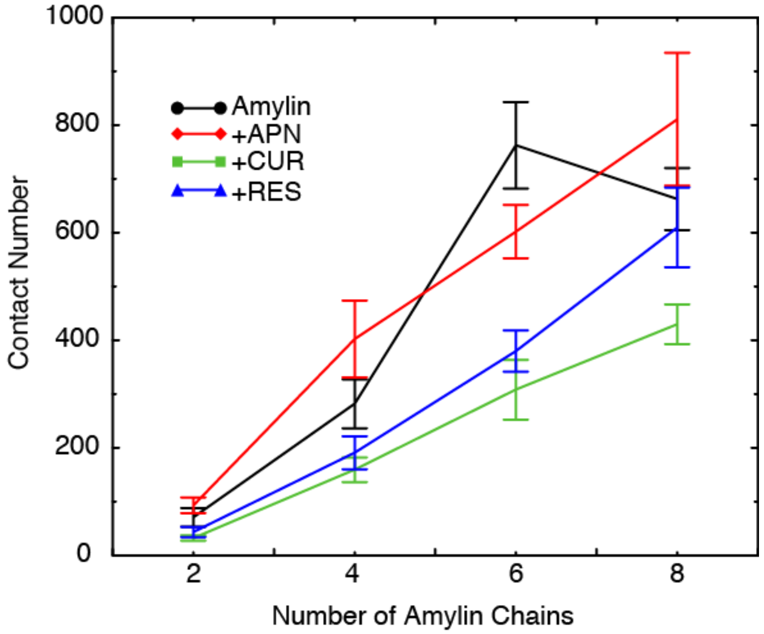

Figure S4. Amyloidogenic contact numbers. Average inter-peptide contact made by the amyloidogenic segment (residues 22–29) of IAPP in different simulation systems. The number of IAPP chains is varied from 2 to 8. Amyloidogenic contact number, like the total contact number (Fig. 2A), is generally lower in the presence of curcumin and resveratrol while aspirin seems to have no significant effect on IAPP aggregation.

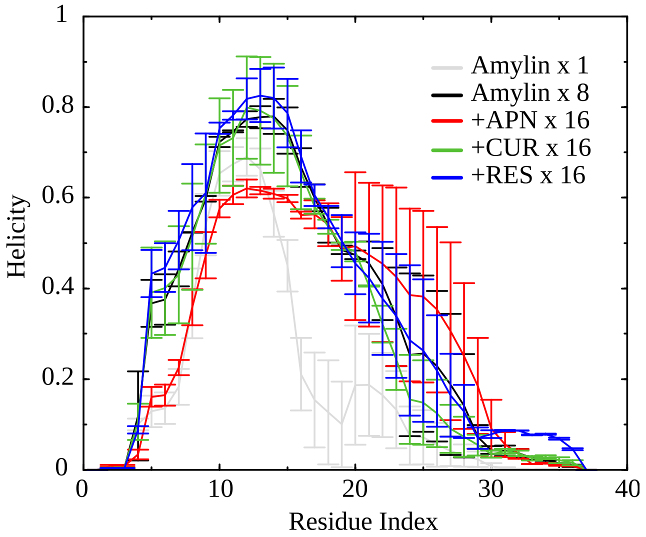

Figure S5. Helical structure propensities of IAPP residues in octamer simulations in the presence of small molecules. The helicities of IAPP monomer and octamer (with no small molecules) are shown for comparison. The helicity of IAPP aggregates is considerably higher than monomer IAPP. The error bars correspond to standard deviations from independence simulations.

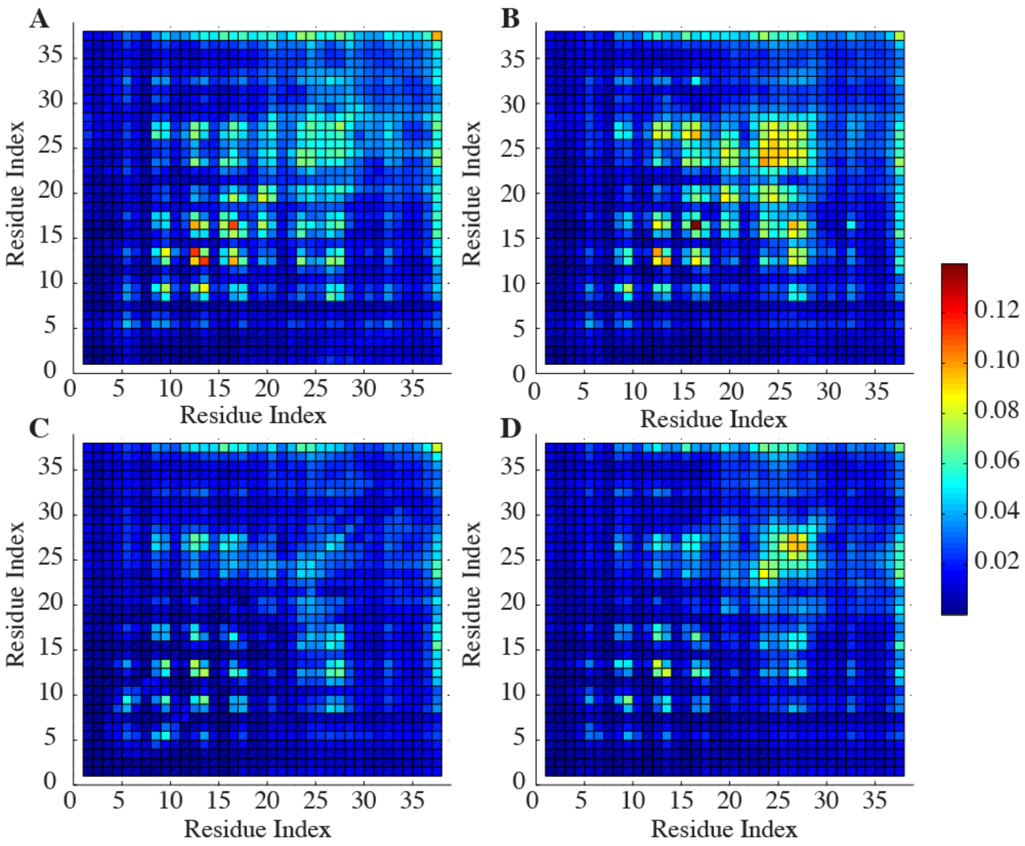

Figure S6. The quaternary contacts of amino acids. The average inter-chain contact frequencies between all pair-wise residues were computed from octamer simulations. Each pair-wise contact frequency was normalized by the number of chains and averaged over time. The panels correspond to results from summations without small molecules (A), and with aspirin (B), curcumin (C) and resveratrol (D). The inter-chain contacts between Cα atoms with a cutoff distance of 7.5 Å were used to calculate the contact numbers. The numbers were normalized by the number of IAPPs.

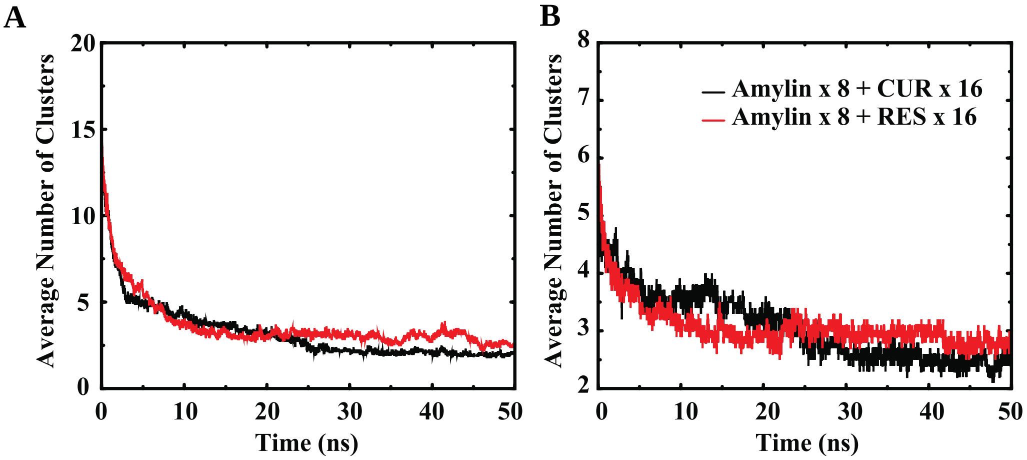

Figure S7. Time dependence of cluster properties. (A) The average number of clusters containing IAPP chains in the presence of CUR and RES small molecules. (B) The average number of CUR and RES containing clusters in the same set of simulations. These plots plateau after about 25 ns.

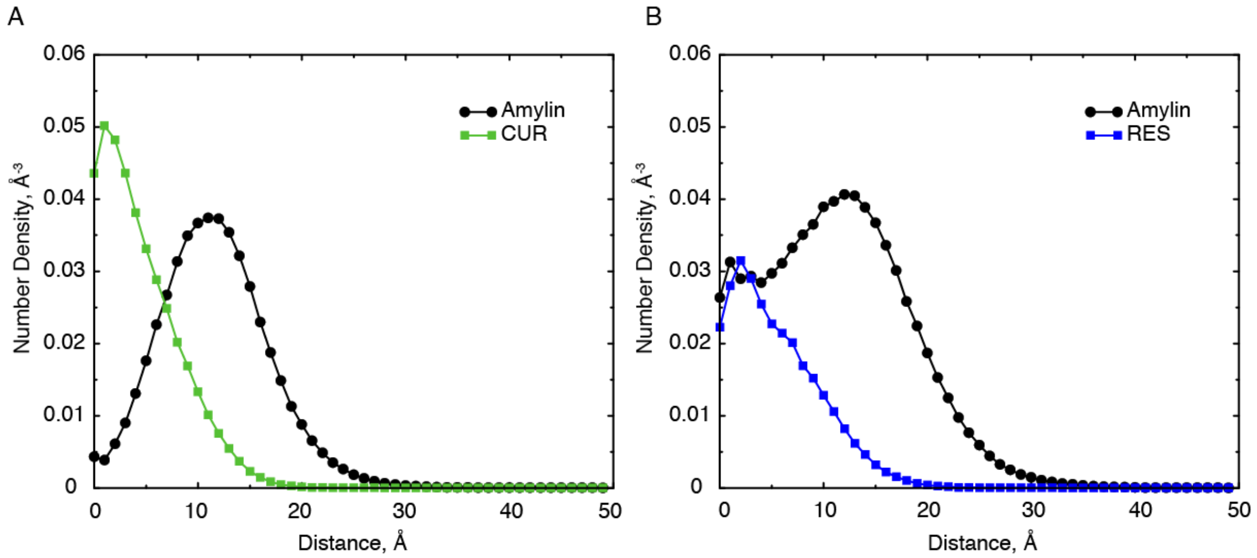

Figure S8. IAPP-small molecule cluster properties. Densities of polyphenol and IAPP atoms as functions of the distance from the center of mass of their clusters, *R*. We used a bin size of d*R* = 1 Å and computed the corresponding atomic density within two concentric spheres of radii *R* and *R*+d*R*. The atomic density profile was averaged over the ensemble of oligomers from DMD simulations. The IAPP atom densities are shown in black and the polyphenol densities in green (A, curcumin) or blue (B, resveratrol).

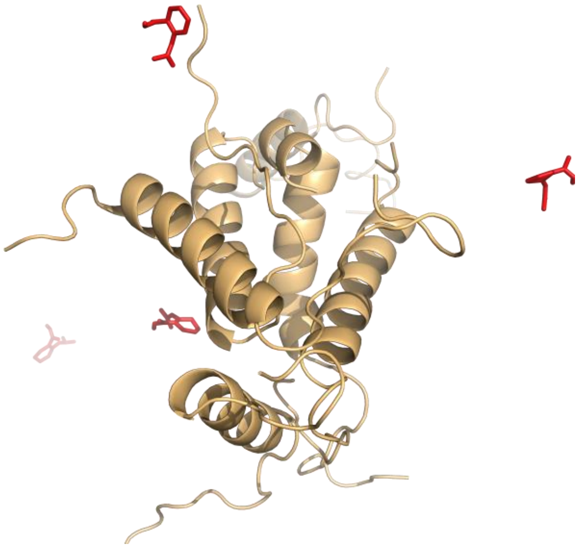

Fig. S9. The pentamer structure in IAPP-aspirin system. A typical snapshot structure of an IAPP pentamer formed during the course of DMD simulation. Unlike in the case of curcumin and resveratrol, aspirin molecules do not aggregate and form a core; instead they are dominantly solvated and only bind on the surface of the oligomer.
